# Experimental Protoporphyria: Effect of Bile Acids on Liver Damage Induced by Griseofulvin

**DOI:** 10.1155/2015/436319

**Published:** 2015-04-07

**Authors:** María del Carmen Martinez, Silvina Fernanda Ruspini, Susana Graciela Afonso, Roberto Meiss, Ana Maria Buzaleh, Alcira Batlle

**Affiliations:** ^1^Departamento de Química Biológica, Facultad de Ciencias Exactas y Naturales, Universidad de Buenos Aires, Ciudad Universitaria, Intendente Güiraldes 2160, 1428 Buenos Aires, Argentina; ^2^Centro de Investigaciones sobre Porfirinas y Porfirias, CONICET-UBA, Avenida Córdoba 2351, 1120 Buenos Aires, Argentina; ^3^Departamento de Patología, Instituto de Estudios Oncológicos, Academia Nacional de Medicina, Pacheco de Melo 3081, 1425 Buenos Aires, Argentina

## Abstract

The effect of bile acids administration to an experimental mice model of Protoporphyria produced by griseofulvin (Gris) was investigated. The aim was to assess whether porphyrin excretion could be accelerated by bile acids treatment in an attempt to diminish liver damage induced by Gris. Liver damage markers, heme metabolism, and oxidative stress parameters were analyzed in mice treated with Gris and deoxycholic (DXA), dehydrocholic (DHA), chenodeoxycholic, or ursodeoxycholic (URSO). The administration of Gris alone increased the activities of glutathione reductase (GRed), superoxide dismutase (SOD), alkaline phosphatase (AP), gamma glutamyl transpeptidase (GGT), and glutathione-S-transferase (GST), as well as total porphyrins, glutathione (GSH), and cytochrome P450 (CYP) levels in liver. Among the bile acids studied, DXA and DHA increased PROTO IX excretion, DXA also abolished the action of Gris, reducing lipid peroxidation and hepatic GSH and CYP levels, and the activities of GGT, AP, SOD, and GST returned to control values. However, porphyrin accumulation was not prevented by URSO; instead this bile acid reduced ALA-S and the antioxidant defense enzymes system activities. In conclusion, we postulate that DXA acid would be more effective to prevent liver damage induced by Gris.

## 1. Introduction

Erythropoietic protoporphyria (EPP) is a hereditary disease due to a decreased activity of ferrochelatase (Fech) (EC.4.99.1.1), the last enzyme of heme pathway catalyzing the incorporation of ferrous iron (II) into protoporphyrin IX (PROTO IX) to synthetize heme [1, 2]. EPP patients show clinical manifestations attributed to the excessive accumulation of PROTO IX, mainly in erythrocytes, liver, and skin. All symptomatic patients show photosensitivity because of the presence of PROTO IX in the skin that generates free radicals after its activation by light [3, 4]. A few EPP patients develop chronic liver disease [5]. There is a correlation between liver damage and PROTO IX concentration in erythrocytes which in some cases is so severe that it could require liver transplantation [6, 7].

PROTO IX is excreted by the hepatobiliary-fecal route and can move to the enterohepatic recirculation. The entrance of PROTO IX to the liver is not saturated; when its excretion through the bile fails to remove the excess of PROTO IX produced by the bone marrow and the liver, this porphyrin accumulates in hepatocytes [8].

Bile acids have choleretic and digestive actions. In humans, bile salts are used as a cathartic to promote bile flow after biliary surgery and to reverse the formation of calculi. In EPP patients bile acids have been used to increase liver porphyrins excretion enhancing cholestasis and in the established cirrhosis to improve both cholestasis and liver function [9–11].

The antifungal griseofulvin (Gris) develops in animals a model of EPP [12] with liver manifestations [13, 14]. Gris is metabolized through cytochrome P450 (CYP) producing N-methyl PROTO IX a potent inhibitor of Fech enzyme [15, 16]. When administrating Gris to mice, several morphological and biochemical hepatic changes were observed, as a result of a marked inhibition of Fech and the reduction of the heme regulatory pool that, secondarily, stimulates the enzyme *δ*-aminolevulinic acid synthetase (ALA-S). To porphyrin accumulation followed cellular damage, inflammatory and necrotic events [17], resembling human EPP associated with liver failure. The high concentration of porphyrins, known generators of reactive oxygen species (ROS), affects hepatic redox balance and, consequently, the antioxidant defense enzymes system. In animals treated with Gris, we observed an increased activity of the enzymes glutathione reductase (GRed), superoxide dismutase (SOD), and glutathione-S-transferase (GST) and high levels of reduced glutathione (GSH) and malondialdehyde (MDA), while the activities of glutathione peroxidase (GPx) and catalase were reduced [17]. Gris also induces bile flow reduction as a result of liver damage, leading to the hepatocytes accumulation of toxic endogenous bile acids such as taurocholic and taurodeoxycholic acid [18].

The main difference between human EPP and Gris animal model is that the overproduction of PROTO IX in the latter begins in the liver instead of the bone marrow [3, 19].

Considering the damage produced by Gris in mice liver, the aim of this study was to investigate the effect of administrating deoxycholic (DXA), dehydrocholic (DHA), chenodeoxycholic (CHENO), and ursodeoxycholic (URSO) acids on heme metabolism, antioxidant defense enzymes system, and CYP levels in Gris treated mice, to assess whether they were able to accelerate the excretion of porphyrins through the bilis, in an attempt to diminish liver damage.

## 2. Materials and Methods

### 2.1. Animals

Male mice* CF1* weighing 15–17 g at the start of intoxication were used. They were maintained in controlled conditions and allowed free access to food (Purina 3, Asociación de Cooperativas Argentinas, San Nicolás, Buenos Aires, Argentina) and water. A 12/12 h light/dark cycle was kept. Animals received human care and were treated in accordance with the guidelines established by the Committee of the Argentine Association of Specialists in Laboratory Animals Care (AADEALC).

### 2.2. Experimental Design

All treatments were performed at the same time of the day. In each experiment, animals were separated into 4 groups of 6 mice each and during two weeks they received the drugs as follows: group I: control diet (standard diet supplemented with corn oil, 10 mL/100 g); group II: control diet plus Gris (0.5% w/w; 280 mg/mice/2 weeks); group III: control diet plus DXA (0.33%; 231 mg/mice/2 weeks) in the drinking water or CHENO (0.01% w/w; 6 mg/mice/2 weeks), DHA (0.33% w/w; 190 mg/mice/2 weeks), or URSO (0.2% w/w; 112 mg/mice/2 weeks) in food: group IV: control diet plus Gris (0.5% w/w; 280 mg/mice/2 weeks) and DXA (0.33%; 231 mg/mice/weeks) in the drinking water or CHENO (0.01% w/w; 6 mg/mice/2 weeks), DHA (0.33% w/w; 190 mg/mice/2 weeks), or URSO (0.2% w/w; 112 mg/mice/2 weeks) in food. Animals were starved 16 hours before sacrifice under ether anaesthesia.

### 2.3. Tissue Preparation

Blood was obtained by cardiac puncture and plasma was separated by centrifugation. The faeces of each cage were collected during the 16 hours before sacrifice. Liver was removed and immediately processed. Experimental conditions for liver homogenate preparations were different, depending on the parameter assayed. A fraction of the liver was immediately homogenized (1 : 3, w/v) in a solution containing 0.9% NaCl, 0.1 mM Tris HCl pH 7.4, and 0.5 mM EDTA, for ALA-S activity determination. The remainder of the organ, previously perfused with sterile ice cold saline, was removed. A fraction was homogenized (1 : 3, w/v) in ice cold 0.25 M sucrose. After differential centrifugation of this homogenate, supernatant of 10,000 ×g was used for measuring GSH levels and GST activity. An aliquot of this supernatant was then centrifuged at 105,000 ×g for 60 min. The supernatant obtained was used for measuring SOD activity and the pellet for determining total CYP levels. Another fraction of the perfused liver was homogenized (1 : 10, w/v) in 0.05 M sodium phosphate buffer, pH 7.4, and it was used as such for the determination of MDA or it was centrifuged at 10,000 ×g for 10 min; in this supernatant GRed activity was measured. The other fraction of the perfused liver (200–300 mg) was used for quantifying PROTO IX levels. Porphyrins were extracted from liver using the method described by De Matteis and Lim [20] and from faeces according to the method of Lockwood et al. [21].

### 2.4. Biochemical Assays

ALA-S activity was determined following the method of Marver et al. [22]. Lipid peroxidation was estimated as MDA levels using the method of Ohkawa et al. [23]. GSH was quantified according to Rossi et al. [24]. GST, GRed, and SOD activities were determined by the methods of Habig et al. [25], Pinto and Bartley [26], and Paoletti et al. [27], respectively. Total CYP levels were measured according to Omura and Sato [28] and protein concentration was measured by the procedure of Lowry et al. [29]. Plasma activities of AP and GGT were determined using a kit from Wiener lab. (Rosario, Argentina). PROTO IX was quantified fluorometrically (*λ*_ex_ 400 nm, *λ*_em_ 632 nm). Enzyme units were defined as the amount of enzyme that catalyzes the formation of 1 nmol of product under the standard incubation conditions. One unit of SOD is defined as the amount of enzyme causing 50% inhibition on the rate of NADH oxidation measured in the control. Specific activities were expressed as units/mg protein.

### 2.5. Histology and Immunohistochemistry

Livers were removed and fixed in 10% neutral-buffered formalin. Samples of each hepatic lobule were processed routinely and embedded in paraffin. At least six microtome sections of 3–5 *μ*m were stained with Haematoxylin and Eosin. Immunohistochemistry for heme oxygenase (HO1) detection was performed using the streptavidin-biotin-peroxidase complex system LSAB (DAKO) as previously reported [17].

### 2.6. Statistical Analysis

All data represent mean values ± standard deviation of all experiments performed in duplicate. The differences between groups were determined by the analysis of the variance (ANOVA) and the significance level was verified by the Bartlett test. A probability level of 99.9% or 99.5% was considered as significant difference between groups.

## 3. Results

### 3.1. Histology and Immunohistochemistry

**Figure 1:**
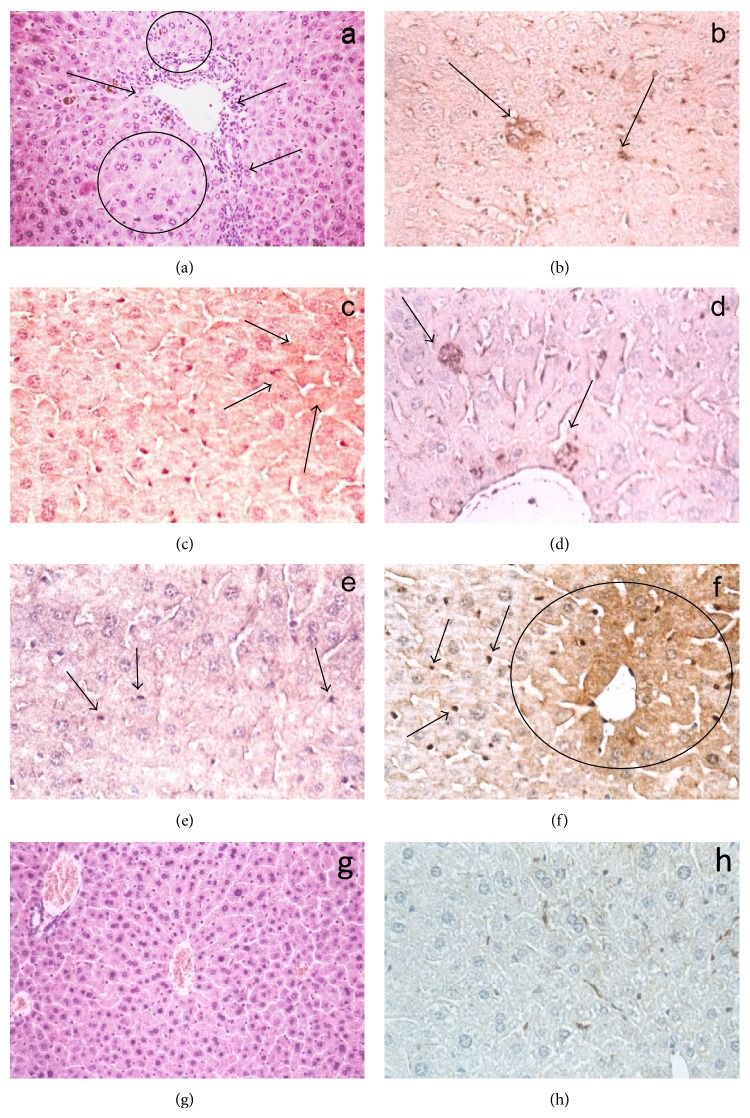
Effect of bile acids on liver histology and HO1 immunostaining. (a) Gris (0.5% w/w) histology showing: liver plates disrupted and distorted; hepatocellular degeneration with small foci of necrosis (circles); ductular proliferation; portal chronic infiltrate (arrows) and cytoplasmic and canalicular cholestasis. HO1 immunostaining: (b) Gris (0.5% w/w) (arrows); (c) Gris (0.5% w/w) plus DHA (0.33% w/v) (arrows); (d) Gris (0.5% w/w) plus CHENO (0.01% w/w) (arrows); (e) Gris (0.5% w/w) plus DXA (0.33% w/w) positive isolated hepatocytes and Kupffer's cells (arrows); (f) Gris (0.5% w/w) plus URSO (0.2% w/w) positive peri centrilobular vein hepatocytes (circle) and Kupffer's cells (arrows); (g) control; (h) control HO1 immunostaining. A unique photo of controls was shown because there were no differences between this group and the groups only treated with the bile acids. Experimental details are described in Section 2.

Histological studies were performed in livers of mice, receiving 0.5% Gris, alone and with the bile acids during 14 days. In all cases, similar alterations were observed (Figure 1(a), control: Figure 1(g)). The liver plates tend to become disrupted and distorted. There is an activation of sinusoidal lining cells with prominent Kupffer's cells. Hepatocellular alterations, predominantly ballooning or feathery degeneration, small foci of necrosis of isolated hepatocytes, and acidophilic bodies, few in numbers, can be seen. The portal changes are ductular proliferation, periductal edema, and portal inflammation. The ductular proliferation occurs along the margins of the portal triad and the inflammatory infiltrate is predominantly lymphocytic with some mononuclear cells. Cytoplasmic and canalicular cholestasis with the presence of bile plug in interlobular ducts can be seen.

The anti-HO1 immunostaining showed positive mark, mainly in isolated hepatocytes, being abundant in Kupffer's cells in liver of Gris or Gris plus bile acid groups (Figures 1(b), 1(c), 1(d), 1(e), and 1(f); control: Figure 1(h)).

### 3.2. Hepatic Damage Markers

**Figure 2:**
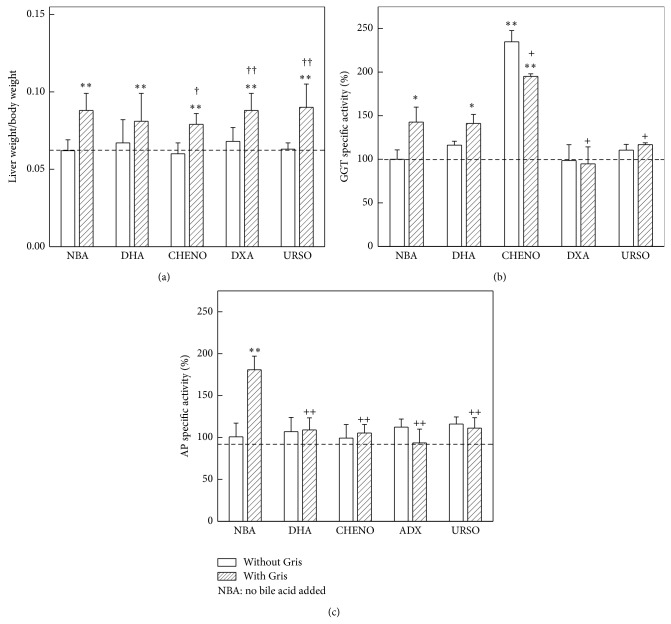
Effect of bile acids on liver/body weight ratio and on enzymes marker of cholestasis. (a) Liver and body weight ratio; (b) GGT, mean control value: 38.97 ± 5.0 *μ*U/mL (*n* = 6); (c) AP mean control value: 421.5 ± 59.7 mU/mL (*n* = 6). Mice received standard diet containing Gris (0.5%, w/w), DHA (0.33% w/w), CHENO (0.01% w/w), DXA (0.33% w/w), URSO (0.20% w/w), or Gris plus one of the bile acids indicated before, during 14 days. Control group received only standard diet in oil (vehicle used for solubilising Gris). Data represent mean values ± S.D. of 6 mice. Results in Figure (b) and (c) are expressed as percentage of the corresponding control value taken as 100%. ^(∗∗)^ *P* < 0.01 significance of differences between groups treated and the control group. ^(†)^ *P* < 0.05 and ^(††)^ *P* < 0.01, significance of differences between the group treated with Gris plus bile acid with respect to the group receiving only bile acid. ^(+)^ *P* < 0.05 and ^(++)^ *P* < 0.01, significance of differences between the group treated with Gris plus bile acid with respect to the group that received only Gris. Other experimental details are described in Section 2.

No significant alterations were detected in liver weight/body weight ratio when DHA, DXA, URSO, or CHENO was administered to control animals. Gris increased 40% (*P* < 0.01) this parameter but this enhancement was not reversed by any of the acids assayed (Figure 2(a)).

Gris induced 40% (*P* < 0.05) GGT activity (Figure 2(b)). When DHA, ADX, or URSO was given to control mice, no significant differences in GGT activity were observed. However, CHENO increased 130% (*P* < 0.01) this enzyme activity; such enhancement also occurred in animals receiving CHENO concomitantly with Gris. This induction was significantly greater than that produced by Gris alone. Administration of DHA did not modify the 40% augmented activity due to Gris. In contrast, DXA and URSO significantly prevented (*P* < 0.05) the induction of GGT caused by Gris, returning to basal levels (Figure 2(b)).

All bile acids assayed prevented the increase of AP activity (80%, *P* < 0.01) produced by Gris (Figure 2(c)).

### 3.3. Heme Biosynthesis

**Figure 3:**
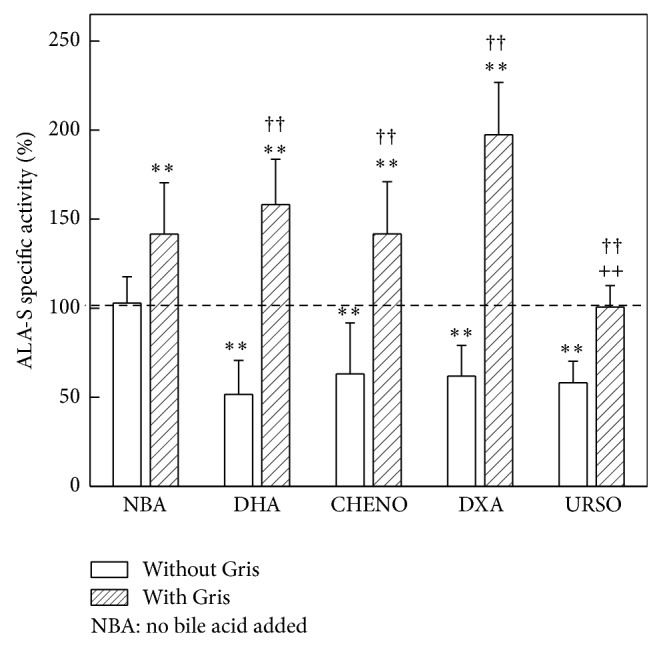
Effect of bile acids on ALA-S activity. Results are expressed as percentage of the corresponding control value taken as 100%. ALA-S activity mean control value: 0.131 ± 0.019 nmol/mg protein (*n* − 6). ^(∗∗)^ *P* < 0.01 significance of differences between groups treated and control group. ^(++)^ *P* < 0.01 significance of differences between the groups treated with Gris plus bile acid with respect to the group receiving only Gris. More experimental details are described in legend of Figure 2.

ALA-S activity was increased 40% (*P* < 0.01) in animals treated with Gris. All the bile acids given alone diminished 40% (*P* < 0.01) ALA-S, but when they were administered with Gris, no effect was observed with respect to those animals receiving only Gris, except in the case of URSO which restored ALA-S activity to basal levels (Figure 3).

The accumulation of PROTO IX in liver increased 50-fold by the action of Gris alone or Gris plus CHENO or URSO; however, DHA or DXA administration reduced around 90% (*P* < 0.01) porphyrin enhancement levels induced by Gris, but without returning to basal levels (Table 1).

Among the bile acids studied, only CHENO increased fecal PROTO IX excretion (3-fold with respect to control group). Instead, Gris increased 400% (*P* < 0.01) PROTO IX excretion; treatments with DHA or DXA produced an excretion 3-fold (*P* < 0.01) higher than that of animals receiving only the antifungal. When Gris was administrated simultaneously with CHENO or URSO, PROTO IX levels in feces were similar to their respective controls (Table 1).

### 3.4. Oxidative Stress

**Figure 4:**
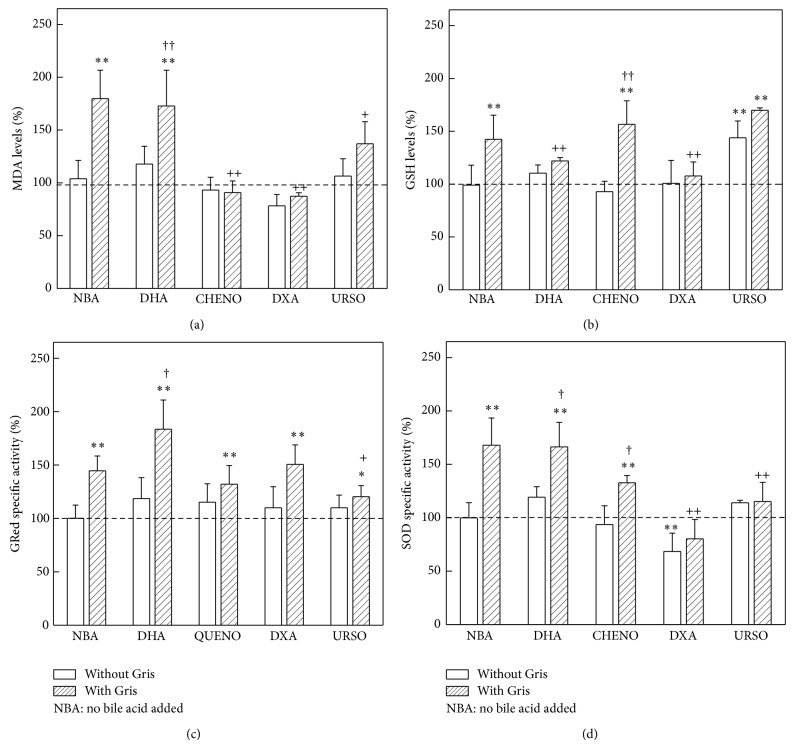
Effect of bile acid on MDA, GSH levels and liver antioxidant defense system. (a) MDA levels, mean control value: 7.94 ± 1.39 nmol/mg protein (*n* − 6). (b) GSH levels, mean control value: 27.05 ± 5.11 nmol/mg protein (*n* − 6). (c) GRed, mean control value: 40.11 ± 4.96 nmol/mg protein (*n* − 4); (d) SOD, mean control value: 71.95 ± 10.93 nmol/mg protein (*n* − 6). Results are expressed as percentage of the corresponding control value taken as 100%. ^(∗)^ *P* < 0.05 and ^(∗∗)^ *P* < 0.01, significance of differences between the groups treated and the control group. ^(+)^ *P* < 0.05 and ^(++)^ *P* < 0.01, significance of differences between the group treated with Gris plus bile acid with respect to that receiving only Gris. ^(†)^ *P* < 0.05 and ^(††)^ *P* < 0.01, significance of differences between the group treated with Gris plus bile acid with respect to the group receiving only bile acid. More experimental details are described in legend of Figure 2.

MDA levels were not changed in control animals receiving only the bile acid assayed. Gris alone or with DHA increased 80% (*P* < 0.01) MDA levels (Figure 4(a)). Whereas URSO dropped 40% (*P* < 0.05) the increase of MDA levels produced by Gris, the treatment with CHENO or DXA returned MDA content to basal levels (*P* < 0.01) (Figure 4(a)).

The administration of URSO alone produced a 40% (*P* < 0.01) enhancement of GSH levels, alike in mice treated with Gris or with the combination of CHENO–Gris or URSO–Gris. DXA and DHA restored GSH to basal levels (Figure 4(b)).

None of the bile acids assayed modified the activity of GRed in control animals. Gris induced 45% (*P* < 0.01) GRed activity. These enhanced levels were not reversed by the administration of CHENO, DHA, or DXA; however addition of URSO decreased to 20% (*P* < 0.01) GRed activity induced by Gris alone (Figure 4(c)).

The administration of DXA to control animals reduced 20% (*P* < 0.01) SOD activity, while no changes were produced by the other bile acids assayed. Gris induced 70% (*P* < 0.01) SOD activity. When animals received DXA or URSO with Gris, this enzyme activity decreased 70% (*P* < 0.01) and 50% (*P* < 0.01), with respect to Gris group, reaching control values (Figure 4(d)).

### 3.5. Drug Metabolizing System

**Figure 5:**
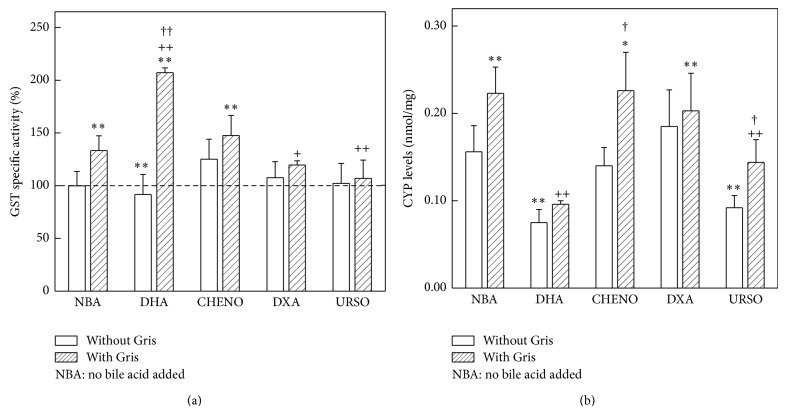
Effect of bile acids on GST activity and on CYP levels. (a) GST, mean control value: 22.77 ± 1.20 *μ*nmol/mg protein (*n* − 6); (b) CYP levels. Results in (a) are expressed as percentage of the corresponding control value taken as 100%; in (b) data are expressed as mean values ± S.D. ^(∗)^ *P* < 0.05 and ^(∗∗)^ *P* < 0.01 significance of differences between group treated and control group. ^(+)^ *P* < 0.05 and ^(++)^ *P* < 0.01, significance of differences between the groups treated with Gris plus bile acid with respect to the group receiving only Gris. ^(†)^ *P* < 0.05 and ^(††)^ *P* < 0.01, significance of differences between the group treated with Gris plus bile acid with respect to the group receiving only bile acid. More experimental details are described in legend of Figure 2.

CHENO administered to control animals increased 25% (*P* < 0.01) GST activity, without any alteration by the action of the other bile acids studied. Gris induced 30% (*P* < 0.01) enzyme activity, remaining enhanced after CHENO plus Gris treatment. DHA produced an additional induction (60% *P* < 0.01) to that induced by Gris. Instead, when DXA or URSO was given to Gris feeding mice, GST activity returned to control values (Figure 5(a)).

DHA and URSO administration to controls animals produced a 40% (*P* < 0.01) decrease of CYP levels, while no changes after DXA or CHENO treatments were observed. Gris leads to an increase of 40% (*P* < 0.01) in hepatic CYP levels, without any modification when Gris was given simultaneously with CHENO or ADX. DHA diminished 130% (*P* < 0.01) the hepatic CYP induction produced by Gris. Only URSO was able to reverse CYP levels to normal values when it was given concomitantly with Gris (Figure 5(b)).

## 4. Discussion

In EPP patients, when hepatocellular damage progresses to a critical stage, PROTO IX accumulation is markedly accelerated due to biliary excretion impairment [30]. EPP patients used bile acids to increase porphyrins excretion and to improve cholestasis [11, 30, 31]. In liver perfusion studies, Avner and Berenson [32] have observed that the limited PROTO IX secretion into bile can be increased by bile salts, supporting the hypothesis that the choleretic bile salts may also increase PROTO IX secretion in protoporphyria.

In animal studies, published results are contradictory; in mice treated with Gris, it was reported that URSO administration may have a cytoprotective and choleretic action on the liver injury induced by Gris [33, 34] although Irifune et al. [35] did not notice any effect after administration of URSO in the same animal model.

In our model, the administration of different bile acids led to several effects. Among the bile acids assayed, only DXA and DHA increased PROTO IX excretion. We have found that URSO did not prevent porphyrins accumulation; however it decreased ALA-S activity, indicating that it is acting on heme biosynthesis. HO1 expression was induced as a consequence of the oxidative stress produced by Gris and PROTO IX accumulation; but this effect was not reversed after bile acids treatment. In our study only URSO and DXA were able to prevent enhancement of GGT and PA induced by Gris, indicating the existence of certain protection against these disturbances, although no histological changes were observed when compared to the group treated only with Gris.

It has been reported that URSO possesses antioxidant properties, while hydrophobic bile acids generate ROS acting as prooxidants [36, 37]. Porphyrins produce ROS and thereby an imbalance in the cell redox status. As expected, in animals treated with DXA and Gris, an increase in PROTO IX clearance leading to a decrease of lipid peroxidation was detected, but this action was not observed when the other bile acids were given. GSH levels remained within control values when DHA and DXA were administered along with Gris. Nevertheless these findings did not reflect the high activity of GRed observed in the animals. However, URSO reduced the induction of GRed without altering GSH levels. In previous studies we have observed that GPx activity significantly decreased in mice treated with Gris, so we might assume that other enzymes were consuming GSH [17]. SOD activity remained within control values in mice treated with URSO, DXA alone or plus Gris.

The increase of GST activity in mice receiving Gris is indicative of liver damage [15]. It has been described that, under oxidative stress, GST is activated by the generation of S-thiolation mediated by hydroperoxides, with GSH being the donor of the SH groups in a reversible reaction, that depends on the intracellular GSH redox balance [38]. So the increase of GST activity observed in our model would be related to the levels of hepatic GSH and ROS [17]. In the presence of cholestasis, Phase I drug metabolizing system plays an important role in both humans [39] and animal models [40, 41]. Tanaka et al. [42] suggested that the decreased CYP levels, after treatment with DXA, could occur due to the presence of cholestasis and could also reflect the toxicity of these bile acids.

We have previously observed an increase of hepatic CYP levels in animals treated with Gris alone [17], but it did not happen when mice were simultaneously treated with Gris and DHA, DXA, or URSO, indicating that these drugs would alter Gris metabolism.

In conclusion, taking into account that DXA effectively reduced porphyrins accumulation induced by Gris and consequently protected the liver against lipid peroxidation and maintaining GSH levels, GGT and AP activities, and drug metabolizing system in basal levels, we postulate that this bile acid would be the most effective in preventing the liver damage induced by Gris.

## Figures and Tables

**Table 1:** Effect of bile acids on PROTO IX levels in liver and feces.

Bile acid	PROTO IX levels			
	Liver (ng/mg protein)		Feces (ng/mg feces)	
	Without Gris	With Gris	Without Gris	With Gris
NBA	0.42 ± 0.04	20.25 ± 3.58^**^	2.22 ± 0.04	10.25 ± 0.42^**^
DHA	0.47 ± 0.07	2.88 ± 0.68^++††^	3.88 ± 0.06	32.12 ± 0.57^++††^
CHENO	0.44 ± 0.06	20.94 ± 2.11^∗∗††^	6.58 ± 0.008	6.85 ± 0.06
DXA	0.43 ± 0.09	1.11 ± 0.20^++††^	2.53 ± 0.004	31.82 ± 0.38^++††^
URSO	0.51 ± 0.07	27.59 ± 3.58^∗∗††^	02.07 ± 0.05	4.55 ± 0.05^∗∗††^

NBA: no bile acid added.

Mice received standard diet containing Gris (0.5%, w/w), DHA (0.33% w/w), CHENO (0.01% w/w), DXA (0.33% w/w), URSO (0.20% w/w), or Gris plus one of the bile acids, over 14 days. Control group received only standard diet in oil (vehicle used for solubilising Gris). Data represent mean values ± S.D. of 6 mice. ^**^ *P* < 0.01: significant difference between the groups treated with respect to the control group. ^++^ *P* < 0.01 significance of differences between the group treated with Gris plus bile acid with respect to the group that received only Gris. ^††^ *P* < 0.01: significant difference between the group treated with Gris plus bile acid with respect to the group treated only with bile acid. Other experimental details are described in Section 2.

## Abbreviations

ALA-S: *δ*-Aminolevulinic acid synthetase
AP: Alkaline phosphatase
CHENO: Chenodeoxycholic acid
CYP: Cytochrome P450
DHA: Dehydrocholic acid
DXA: Deoxycholic acid
EPP: Erythropoietic protoporphyria
Fech: Ferrochelatase
GGT: Gamma glutamyl transpeptidase
GPx: Glutathione peroxidase
GRed: Glutathione reductase
Gris: Griseofulvin
GSH: Reduced glutathione
GST: Glutathione-S-transferase
HO1: Heme oxygenase
MDA: Malondialdehyde
ROS: Reactive oxygen species
PROTO IX: Protoporphyrin IX
S.D.: Standard deviation
SOD: Superoxide dismutase
URSO: Ursodeoxycholic acid.
